# Modulator-free quadrature amplitude modulation signal synthesis

**DOI:** 10.1038/ncomms6911

**Published:** 2014-12-19

**Authors:** Zhixin Liu, Joseph Kakande, Brian Kelly, John O’Carroll, Richard Phelan, David J. Richardson, Radan Slavík

**Affiliations:** 1 Optoelectronics Research Centre, University of Southampton, Southampton SO17 1BJ, UK; 2 Bell Labs, Alcatel-Lucent, Holmdel, New Jersey 07733, USA; 3 Eblana Photonics Inc., Unit 31, Pearse Street, Dublin 2, Ireland

## Abstract

The ability to generate high-speed on–off-keyed telecommunication signals by directly modulating a semiconductor laser’s drive current was one of the most exciting prospective applications of the nascent field of laser technology throughout the 1960s. Three decades of progress led to the commercialization of 2.5 Gbit s^−1^-per-channel submarine fibre optic systems that drove the growth of the internet as a global phenomenon. However, the detrimental frequency chirp associated with direct modulation forced industry to use external electro-optic modulators to deliver the next generation of on–off-keyed 10 Gbit s^−1^ systems and is absolutely prohibitive for today’s (>)100 Gbit s^−1^ coherent systems, which use complex modulation formats (for example, quadrature amplitude modulation). Here we use optical injection locking of directly modulated semiconductor lasers to generate complex modulation format signals showing distinct advantages over current and other currently researched solutions.

## Abstract (web summary)

Quadrature amplitude modulation signalling is currently enabling rapid data transfer capacity growth, but it still has associated drawbacks. Here, Liu *et al.* use optical injection locking to generate complex modulation format signals with reduced consumption, small footprint and easy integration.

## Graphical abstract

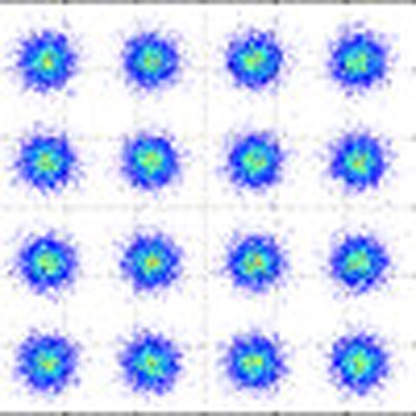

Direct modulation of a laser^1^ has always been used in optical telecommunications. Although distance–capacity product capabilities using this approach have been limited, it represents a very cost-effective solution, making it a prime choice especially when transmission distance is limited, for example, within a data centre or a supercomputer (hundreds of thousands within a single supercomputer) or in ‘last-mile’ telecommunications. For long-distance or high-capacity transmission, however, digital optical signals are generated using a continuous-wave (CW) laser source and an external Mach–Zehnder modulator^2^. To respond to the ever-increasing requirements on data transfer capacity, on–off-keyed signalling is gradually being replaced by quadrature amplitude modulation (QAM) signalling^3^ that enables increasing the capacity without the need to scale up the bandwidth of the electrical parts of the system, which is both costly and power hungry^3^. Nevertheless, QAM signalling requires in-phase-quadrature (IQ) modulator structure that is more complicated than on–off-keyed and places more stringent requirements on the modulator performance. Modulators based on the electro-optic effect (such as the industry-standard LiNbO_3_-based devices) offer superior performance relative to modulators exploiting charge-carrier effects in semiconductors, which, although capable of generating larger refractive index changes, also inherently impart undesirable amplitude modulation. Although LiNbO_3_-based modulators offer unparalleled performance, they are long (centimetre-scale), expensive and difficult to integrate with the laser, making the dense integration of tens to hundreds of transmitters as now required challenging. Consequently, major effort is being directed to obtain equivalent performance using other materials for the modulator including InP^4^,^5^, GaAs^6^ and silicon^7^. However, besides parasitic residual amplitude modulation, these modulators suffer from high propagation loss, allowing only for short devices requiring large radio frequency (RF) drive voltages. Nevertheless, through intensive research, devices generating QAM signals have recently appeared enabled both by improved modulator design and the use of digital signal processing (DSP) at both transmitter and receiver to compensate for the non-ideal phase and amplitude transfer functions. The current state of the art includes: a 40-GBaud InP modulator delivering 160 Gbit s^−1^ via external polarization multiplexing of two QPSK (quadruple phase shift keying)^4^ signals, an InP-based photonic integrated circuit (laser+modulator integrated) capable of 256-Gbit s^−1^ operation using 32-GBaud polarization-multiplexed 16QAM^5^; a GaAs-based modulator at 150 Gbit s^−1^ using 25-GBaud QAM signals^6^; and finally a silicon-based modulator capable of 224 Gbit s^−1^ 16QAM^7^. Although the progress has been impressive, several drawbacks still remain. Specifically, the number of high-speed RF connections to the modulator is twice that ideally is needed due to the requirement for push-pull operation (increasing packaging cost and power requirements); the required RF power is high; computationally heavy DSP is needed—leading to a requirement for high effective number of bits and high bandwidth converters.

It is worth mentioning that other IQ modulator configurations have also been reported, for example, using four amplitude modulators^8^ and more complex configurations based on a greater number of phase modulators that relax the requirements on the RF drive signals^9^ or improve the linearity of the modulator response^10^.

Here we use a combination of optical injection locking (OIL) and direct modulation of semiconductor lasers to generate IQ-modulated optical signals. Our approach allows for all the benefits of direct modulation, including high linearity, reduced power consumption, small footprint and ease of integration, and opens up a promising new route to the realization of cost-effective, high-performance monolithically integrated QAM transmitters. However, the impact of this work can be seen far beyond telecommunications. Directly current modulated lasers are devices of huge commercial relevance and are widely used in many applications, for example, optical sensing and high-power fibre lasers. In these fields, the inability to accurately control the full optical field through direct current modulation is a fundamental problem, limiting applications to just those requiring intensity modulation and constraining broader use. The new capability we demonstrate here could have a significant impact within the many scientific and engineering communities that are directly concerned with or exploit laser radiation. For example, the coherent combination of high-power lasers could greatly benefit from the control of amplitude and phase that we have demonstrated from low-cost semiconductor devices and may open the way to significantly higher powers and useful functionalities (for example, beam steering), which require precise control of the phase properties of the combined beams. Another example lies in the photonic generation of arbitrary THz signals as needed in a wide variety of emerging ultrafast applications. A final example is laser radar where complex beam modulation is used to provide high-resolution ranging information.

## Results

### Principle of our IQ transmitter

**Figure 1:**
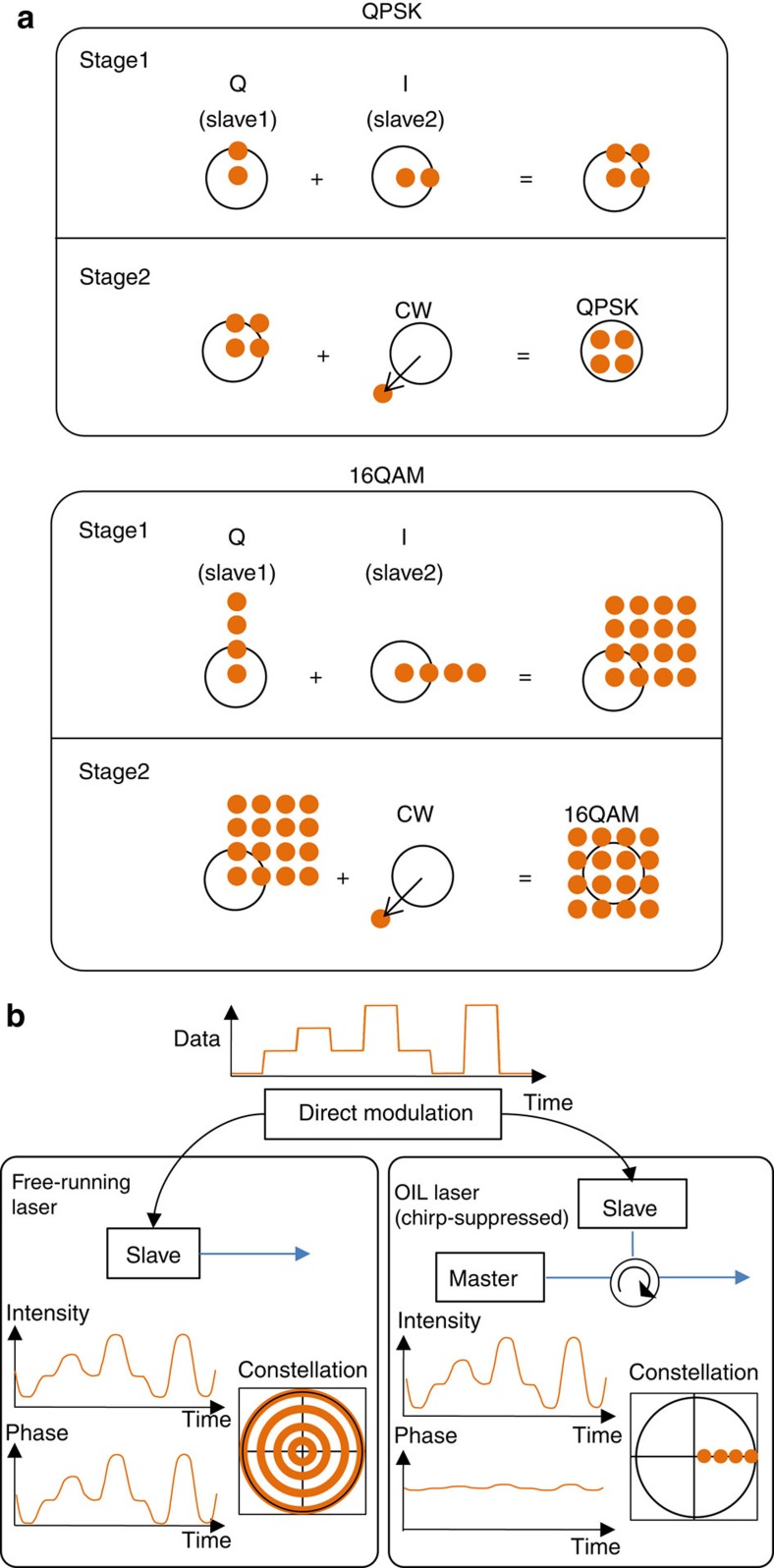
Proposed transmitter based on directly modulated lasers. (**a**) Principle of operation of our transmitter shown using constellation diagrams (in the complex plane) to generate QPSK and 16-level QAM (16QAM) signals. It uses amplitude modulation as the basic building block. Two amplitude-modulated signals are combined (Stage 1), making a signal with a constellation similar to that desired. By removing the carrier component of the signal via interference with the master signal (Stage 2), the desired modulation format signal is obtained. This method allows for an arbitrary level of carrier signal to be present at the output, which is difficult to achieve with a traditional IQ modulator without inducing signal distortion. (**b**) A sketch explaining the difference between a free-running and OIL assisted directly modulated semiconductor laser. Due to the large chirp associated with the directly modulated laser, the constellation consists of four rings rather than four points, making it unsuitable for IQ modulation. However, OIL causes the four circles to ‘collapse’ into four distinct points in the IQ plane.

Schematics of our method^11^,^12^,^13^ showing how to build QAM signals from directly amplitude-modulated (DM) lasers are sketched in Fig. 1a for two specific exemplar signals (QPSK and 16QAM), although we emphasize that the approach can readily be adapted to work for arbitrary IQ modulation. First, we start with amplitude modulation (two levels for QPSK and four levels for 16QAM; Fig. 1a). By combining two such signals with a 90° relative phase shift, we obtain QPSK and 16QAM constellations, albeit not centred at zero (due to the residual carrier). We remove the carrier via destructive interference with an unmodulated component of the carrier (Fig. 1a), finally obtaining ideal QPSK and 16QAM signals.

There are two main obstacles that need to be overcome to realize a DM laser-based ‘IQ Transmitter’ based on the principle shown in Fig. 1a. First, a directly modulated semiconductor laser produces a large frequency chirp^14^ that can be represented as circles rather than points in a constellation diagram (Fig. 1b). The second obstacle in realizing the concept is that the two independent lasers carrying the independent data streams must be coherently combined (with 90° phase shift in our case, Fig. 1a) and thus must be mutually coherent.

We have addressed both issues using an effect known as OIL^15^,^16^. In OIL, a signal from a ‘Master’ laser (in our case a CW laser) is injected into the cavity of a ‘Slave’ laser (a directly modulated laser in our case; Fig. 1a). Under certain conditions, the slave phase locks to the master, even when directly modulated. The bandwidth (that is, difference in frequency of the free-running lasers) over which this locking occurs is called the OIL bandwidth^17^. Once the directly modulated slave is locked to a CW master, its chirp is suppressed (Fig. 1b)^18^,^19^ and its modulation bandwidth can be significantly increased (for example, up to 80 GHz^20^). Moreover, using the same master for two slave lasers allows mutual coherence between the three devices to be established and hence for the stable coherent superposition (interference) of their outputs. This mutual coherence is critical in our transmitter—it allows us to combine the two directly modulated slaves with a 90° shift, as well as to provide carrier suppression through destructive interference with a component of the master signal.

### Experiment

**Figure 2:**
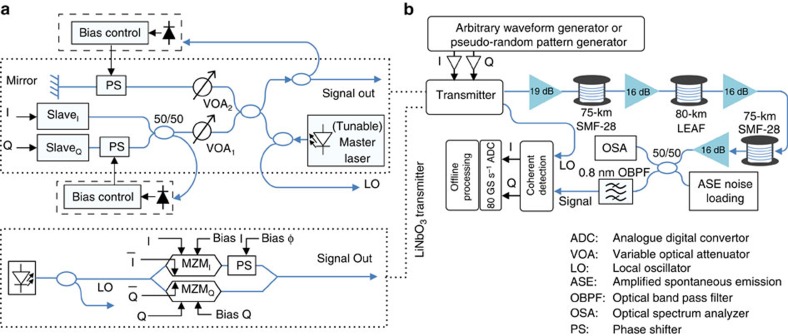
Experimental implementation. (**a**) Experimental set-up of our transmitter system used in our proof-of-principle experiments. (**a**) Upper box: physical implementation of our transmitter; (**a**) lower box: conventional LiNbO_3_-based IQ transmitter used for comparative purposes. It is worth noting that our transmitter requires two times less RF data input signals compared with a standard IQ modulator-based system. Due to the good optical performance, only standard DSP was needed at the receiver side, even though the directly modulated lasers used in our demonstration are just simple Fabry–Perot semiconductor lasers and have not at all been optimized for this application. (**b**) Set-up of our transmission experiment.

**Figure 3:**
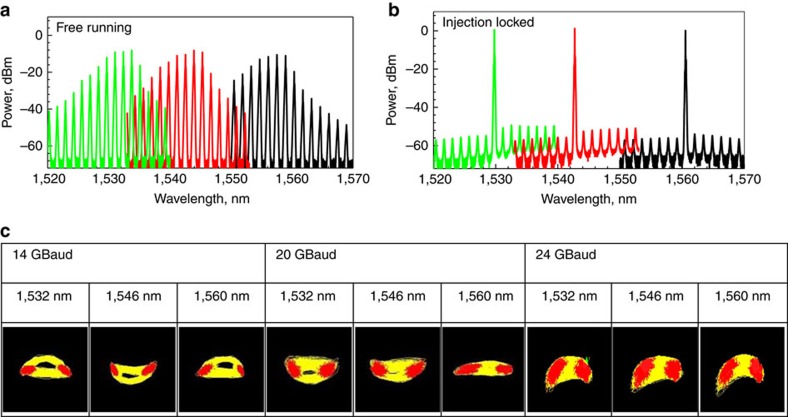
Wavelength tunability of our transmitter. (**a**) Spectra of the free-running slave for three temperature settings. (**b**) Spectra of slave injection locked to a master that was tuned to 1,530 nm (green), 1,543 nm (red) and 1,561 nm (black). (**c**) Eye diagrams for binary phase shift keying modulation (only one slave was on and driven by a two-level data stream) generated at 1,532, 1,546 and 1,560 nm at 14, 20 and 24 GBaud, respectively.

The proof-of-principle set-up for our transmitter is shown in Fig. 2 (described in the Methods section). First, we investigated wavelength tunability. Figure 3a shows the emitted spectra of a free-running slave for three different temperature settings, showing that it can emit light across almost the entire C-band. Under OIL, the laser becomes single mode as shown in Fig. 3b for the three different wavelengths and three slave temperature settings shown in Fig. 3a. By coarse tuning of the slave temperature, OIL can be achieved across the full C-band (1,530–1,560 nm). Figure 3c shows the constellations obtained for binary phase shift keying for various bit rates and wavelengths, showing almost wavelength-independent performance—all were achieved using just one slave laser.

**Figure 4:**
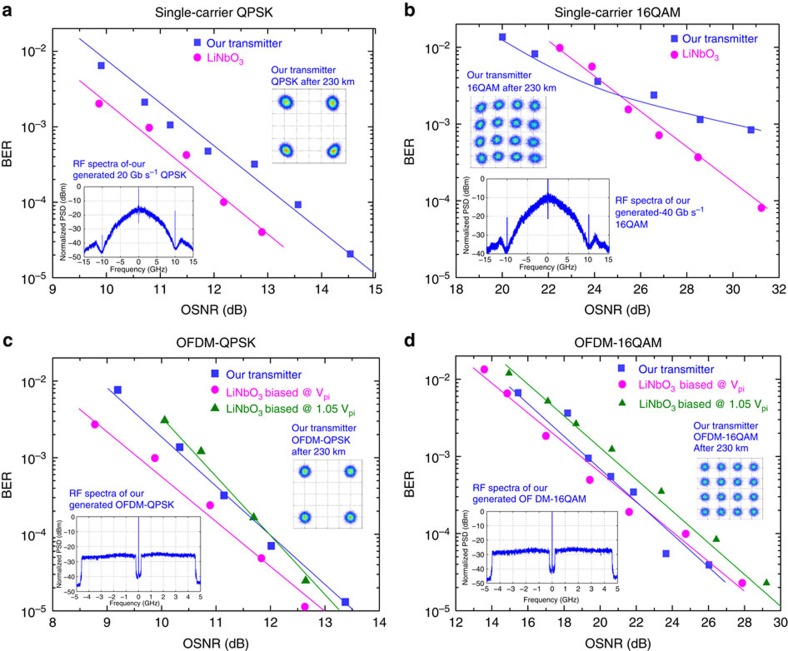
Performance of our transmitter with different modulation formats. (**a**) Single-carrier QPSK format. (**b**) Single-carrier 16-level QAM (16QAM) format. (**c**) OFDM signal with subcarriers encoded using QPSK modulation format. (**d**) OFDM signal with subcarriers encoded using 16QAM modulation formats. RF data signal generated by AWG. Spectra of the generated signal and constellation after the transmission are shown as insets.

**Figure 5:**
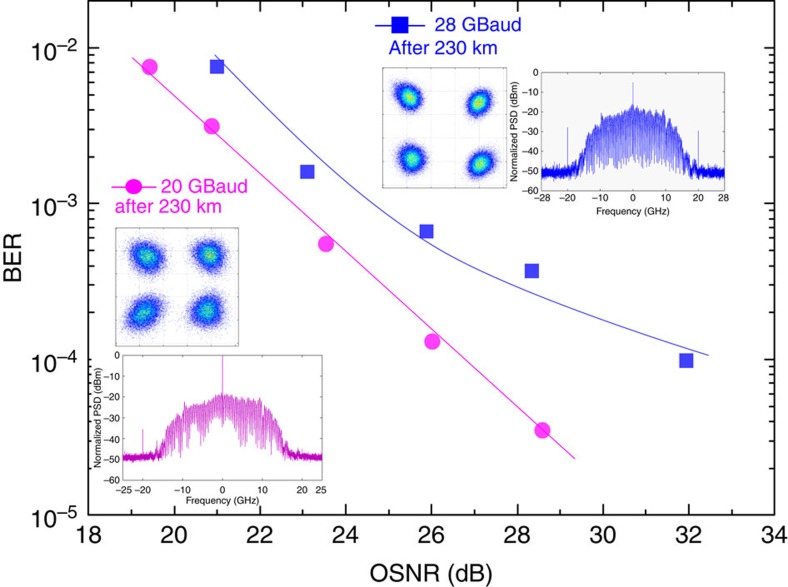
Performance of our transmitter at high baud rates. Single-carrier QPSK data are generated with PRBS RF generator. Spectra of the generated signal and constellation after the transmission are shown as insets. Blue squares: 28 Gbaud; violet circles: 20 Gbaud.

The results of further experiments using an arbitrary waveform generator (AWG) to generate the RF data are shown in Fig. 4a,b (single-carrier QPSK and 16QAM) and Fig. 4c,d (orthogonal frequency-division multiplexed (OFDM) with QPSK and 16QAM). Results obtained with a pseudorandom bit sequence (PRBS) used to generate QPSK at baud rates beyond the capabilities of our AWG (20 and 28 GBaud) are shown in Fig. 5. For direct comparison, we present results obtained with a 22.5-GBaud LiNbO_3_ IQ modulator (Figs 3 and 4).

Figure 4a shows that our transmitter has about 0.8 dB optical signal-to-noise (OSNR) penalty at bit error ratio (BER) of 10^−3^ with respect to our LiNbO_3_ modulator after propagation through 230 km of optical fibre. Figure 4b shows that our transmitter outperforms LiNbO_3_ at high BER (above 2 × 10^−3^), but performs worse for lower BER when the signal is propagated through 230 km of optical fibre. In the constellation (obtained after a dynamic equalization stage), we see a slight distortion, which is a consequence of the (weak) residual chirp present in our transmitter.

In Fig. 4c, we show that our transmitter is capable of generating arbitrary modulated signals by using OFDM. As we see from the spectra (inset), our transmitter shows a linear mapping of electrical signal to optical carrier. The constellation diagrams (obtained after 230 km of fibre propagation and after equalization) show clear constellation clusters and that our transmitter is only slightly inferior to our LiNbO_3_ IQ modulator biased at *V*_*π*_ (0.7- and 0.5-dB OSNR penalty for OFDM-QPSK and OFDM-16QAM, respectively, at a BER of 10^−3^). As OFDM incorporating a component of optical carrier is useful in demodulation, IQ modulators are usually biased slightly away from *V*_*π*_ to obtain the carrier tone^21^. Under the practical assumption of biasing LiNbO_3_ at 1.05*V*_*π*_, our transmitter (which allows the addition of a carrier tone without waveform distortion) actually outperforms LiNbO_3_ for OFDM-16QAM (most probably due to the better linearity of our transmitter, which is discussed in Supplementary Figs 1 and 2), while being very close to it for OFDM-QPSK (Fig. 4d).

Further, we investigated how far in terms of baud rate we can push our transmitter. This proved to be beyond the bandwidth of our AWG, and thus we used a (faster) PRBS generator with two complementary outputs, but which restricted us to work only with QPSK. We found that the slave modulation bandwidth was about 14 GHz, allowing us to go up to 28 GBaud—results for 20 and 28 GBaud are shown in Fig. 5.

## Discussion

As shown above, the performance of our transmitter approaches that of our commercial LiNbO_3_ IQ modulator. In addition, it requires only half the number of high-speed RF inputs with 0.8*V*_pp_ (volts peak-to-peak) drive voltage (as compared with >2*V*_pp_ in LiNbO_3_, InP or GaAs and 5*V*_pp_ in silicon), thereby requiring 25 times less RF power. Similarly to external modulators, our transmitter can be modified to operate in a push-pull mode or using binary-only RF data streams—details are given in Supplementary Figs 3 and 4. Although the lasers in our transmitter require temperature control, we expect that only one temperature controller would be needed if all lasers within the transmitter are integrated into a single integrated photonic circuit. As for competing technologies, they generally require temperature control as well. Although LiNbO_3_ modulators do not themselves require temperature control, the signalling laser usually does. Thus, we conclude that all existing/currently researched solutions require at least one temperature controller. Regarding the power consumption of the various feedback control loops, it is to be appreciated that these are of very low speed ((sub-) kHz speed) and hence they can be designed to consume negligible amounts of power in comparison with the temperature control and RF power consumption. With regards to energetic efficiency, our transmitter has an inherent 3-dB loss as it exploits amplitude modulation in which half of the optical power is within the carrier and that needs subsequently to be removed. The removal process (interference with the master CW laser) requires an identical amount of power from the master laser as is contained in the carrier to be removed, resulting in 5 dB loss (for example, 1 mW of amplitude-modulated signal will become 0.5 mW when the carrier is removed and a further 0.5 mW is needed to remove the carrier. Thus, we need a total of 1.5 mW of optical power to obtain 0.5 mW of carrier-less modulated signal at the output). To obtain the same level of linearity in the modulation response of a LiNbO_3_ modulator as we have in our transmitter, the LiNbO_3_ modulator needs to be operated within its linear response regime only, increasing its loss (typically 2–5 dB) by another 3 dB, making our approach comparable in terms of energetic efficiency to a LiNbO_3_ IQ modulator. In addition, electrical power required to generate the sacrificed carrier power should be significantly smaller than the savings in the high-speed RF power, as our transmitter requires only half of the RF streams.

Although the data rates demonstrated in our current proof-of-principle experiments are relatively modest compared with the most recent results obtained with external IQ modulators, we did so without any optimization of our transmitter components and using only off-the-shelf components designed for other applications. We expect significant improvements when using optimized slave lasers, especially when these are integrated onto a single photonic chip (for example, InP) as we envisage doing. However, even with the present transmitter, we required 25 times lower RF drive power, helped by the fact that we need only half as many RF signals, and obtain a performance reasonably close to the best state-of-the-art modulators. We consider that this makes our transmitter an ideal candidate for Metro and Access networks, where power consumption is the primary concern.

## Methods

### Our transmitter

The slave lasers were a pair of 250-μm long, 1,550-nm AlGaInAs Fabry–Perot lasers’ diode designed for operation at 2.5 Gbit s^−1^, packaged in a commercial high-speed butterfly module with thermo-electric control and an electrical connection bandwidth of 18 GHz. The laser output was directly coupled into a polarization-maintaining (PM) single-mode fibre without any in-built isolator (as needed to allow OIL). The master was either a 40-mW CW telecom-grade C-band tunable laser (used in experiments requiring tunability) or a 10-kHz linewidth fibre laser emitting 20 mW of CW power at 1,555.7 nm. Variable optical attenuator VOA1 attenuated the master laser signal by 3 dB to obtain the optimal injection power of 2 dBm. In an optimized design, VOA1 could be eliminated, thereby requiring less power from the master laser and—at the same time—allowing higher output signal power, as the slave laser output would also not need to pass through this attenuator. The free-running slave laser wavelengths were set (via temperature control) to be slightly higher than the master laser wavelength. The wavelengths were fine-tuned to obtain optimum modulation linearity and minimum residual modulation chirp^22^. The outputs from the two slave lasers were orthogonally combined by setting and maintaining a relative phase shift between their carriers of 90° (that is, operated in quadrature). The relative phase shift between the two slave laser carriers was controlled via a piezoelectric (PZT)-driven fibre stretcher (6-cm long) placed at the output of one of the slave lasers (Fig. 2b). The feedback to keep the two slave lasers in quadrature consisted of a slow photodiode (Fig. 2b) followed by a 1-kHz bandpass filter and a proportional-integral (PI) loop controller. Constructive/destructive interference of the two slaves (corresponding to a relative phase of 0° and 180°, respectively) produced maximum/minimum signal at the photodiode. Hence, in quadrature (90°), the signal at the output of the photodiode changed monotonically with relative phase between the two slave lasers and thus could be used directly as the error signal at the PI controller input. Once the two slave lasers were combined in quadrature, the carrier part of their combination was removed by destructively interfering it with a component of the master laser signal obtained via reflection from a mirror. To keep the destructive interference, another feedback loop was necessary. Again, a slow photodiode followed by a 1-kHz filter, PI loop filter and a PZT stretcher was used. However, unlike in the previous feedback loop, we needed to obtain the phase lock at the minimum of the signal coming out of the slow photodiode (as we operate at the interference minimum). We introduced a small dither signal at the PZT fibre stretcher (15 kHz, producing <1° phase dither), which, in conjunction with lock-in detection, allowed for a suitable error signal. In our experiments, the OIL conditions (injected power, frequency detuning between the free-running master and slave lasers) were set for optimum performance. In practice, these could be maintained by implementing further simple, low-bandwidth phase-lock loops, as we did previously for slave lasers under CW operation^23^. The transmitter set-up is made entirely of PM fibres and PM-coupled components to avoid any polarization drift. Further, to maintain mutual coherence between the three interfering signals (two slaves and a portion of the master), the difference in paths travelled by these three signals was kept to <20 cm, being well below the coherence length of the master laser. Finally, to minimize phase drifts due to environmental factors (temperature, acoustic pick-up), we kept the fibres carrying these signals as physically close together as possible. As a result, feedback with only sub-kHz loop bandwidth was needed to stabilize the path difference as applied via two PZT fibre stretchers. The slave lasers were biased at 43 mA and modulated by electrical signals generated either using a dual-channel AWG, Tektronix AWG7122C with a bandwidth of 9.6 GHz and sampling rate of 10 GS/s or a dual-output PRBS generator capable of operating up to 56 GBaud with a 1-bit output (two levels—OFF and ON). The two RF data streams (two AWG outputs (used as I and Q) or two complementary outputs of a PRBS generator properly delayed to be de-correlated) were amplified by two RF amplifiers to 0.8*V*_pp_ and used to directly drive the two slave lasers. The RF signals sent to the two slave lasers were synchronized using a manual RF phase shifter delay line when the PRBS generator was used, or directly using the AWG. The AWG was used for the generation of the OFDM signals (QPSK and 16QAM), 10-GBaud QPSK and 16QAM (data shown in Figs 3 and 4). When baud rates beyond the AWG capabilities (10 GBaud) were necessary (Figs 2 and 5), the PRBS generator was used, which allowed only for the generation of QPSK signals (having just 1-bit outputs).

### Digital signal processing

In the OFDM experiment, the baseband waveform samples were calculated offline based on a PRBS of 2^17^−1. Both QPSK and 16QAM constellation mapping were employed. An inverse Fourier transform size of 256 was used. Of the 256 subcarriers, 22 high-frequency and 11 low-frequency subcarriers are set to zero, which is common in OFDM as low-frequency channels suffer from a frequency offset penalty and high-frequency channels suffer from the effects of filtering. Eight-sample cyclic prefix is placed before and after each OFDM symbol, resulting in symbol rate of about 36.8 MSymb s^−1^ for each subcarrier. The nominal data rate for QPSK and 16QAM mapping were 16.5 and 32.9 Gbit s^−1^, respectively. The optical carrier is not fully cancelled to generate OFDM with a carrier tone^21^, which results in a carrier-signal ratio of −7 dB. A 16% training overhead was used for symbol synchronization and channel estimation. After 1-tap equalization, the BER was calculated.

In the 10-GBaud single-carrier QPSK and 16QAM experiments, the drive waveforms are differentially encoded using a quadrant rotation algorithm^24^ based on a PRBS of (2^19^−1). At the receiver side, we first compensated for the imperfections in the optical front end (deskew, d.c. block, power imbalance correction and IQ orthogonalization). Then, we downsampled the acquired waveforms to two samples/symbol and compensate the chromatic dispersion using a static equalizer^25^. After that, we used a standard 17-tap constant modulus algorithm (CMA)^25^ and a 22-tap multi-ring CMA^26^ to adaptively compensate the residual impairments for QPSK and 16QAM, respectively. As homodyne detection was used to simplify the experiment, frequency offset compensation was not needed. In the last stage, we compensated the phase noise using a feed-forward carrier recovery algorithm^24^. Finally, the signals were differentially decoded before BER calculation.

For the 20-GBaud and 28-GBaud QPSK experiments, we split the output of a high-speed pseudorandom pattern generator (SHF 12100B) into two branches and introduced a delay to one branch for driving I and Q (PRBS of 2^15^−1). The receiver side processing was similar to that used in the 10-GBaud experiment, except that a 13-tap sliding filter was used to compensate the phase noise^27^.

In the wavelength tunability experiment (Fig. 3), homodyne detection was performed with the help of commercial Agilent DSP algorithms in-built in the Coherent receiver (Agilent DSO-X 93204A+ N4391A).

### Transmission link set-up

The 230-km transmission link incorporated three spans of fibre: 75 km of standard single-mode fibre (SSMF-28) in the first and third span and 80 km of large effective area fibre in the second span. We launched −1 dBm of average power to avoid nonlinearities. At the receiver side, ASE-generated noise loading was used to adjust the OSNR. The OSNR was calculated from the measured signal power and noise floor at 0.1-nm resolution. A 0.8-nm optical bandpass filter was used to filter out the out-of-band noise. A component of CW light (6 dBm) was tapped from the master laser and used as a local oscillator for single-polarization homodyne reception. The polarization of the received signal was manually aligned with that of the local oscillator at the receiver. After coherent detection, the electrical signal was then sampled by a 32-GHz, 80-GS s^−1^ real-time oscilloscope before offline processing.

Most experiments conducted with our transmitter were repeated using a 22.5-GBaud IQ modulator from Fujitsu (FTM7960EX) for comparison purposes. To ensure optimum linearity, *V*_pp_ of the driving RF signals was reduced to ~0.6*V*_*π*_.

## Author contributions

Z.L. designed and carried out all the experiments involving advanced modulation formats. R.S. and J.K. conceived the idea. R.S. built the transmitter and carried out preliminary tests. R.S. led the work. D.J.R. provided overall guidance and support. Z.L., J.K., R.S. and D.J.R. wrote the manuscript. B.K., J.O. and R.P. designed and manufactured the semiconductor slave lasers.

## Additional information

**How to cite this article:** Liu, Z. *et al.* Modulator-free quadrature amplitude modulation signal synthesis. *Nat. Commun.* 5:5911 doi: 10.1038/ncomms6911 (2014).

## Supplementary Material

Supplementary Information Supplementary Figures 1-4
