# Supplementary material for: Modulator-free quadrature amplitude modulation signal synthesis
Source: Nat Commun. 2014 Dec 19;5:5911. doi: 10.1038/ncomms6911 (PMC4284664; doi:10.1038/ncomms6911)
Supplement: Supplementary Information — Supplementary Figures 1-4 [file ncomms6911-s1.pdf]

Linear response comparison of our and $LiNbO_3$ modulator based transmitters

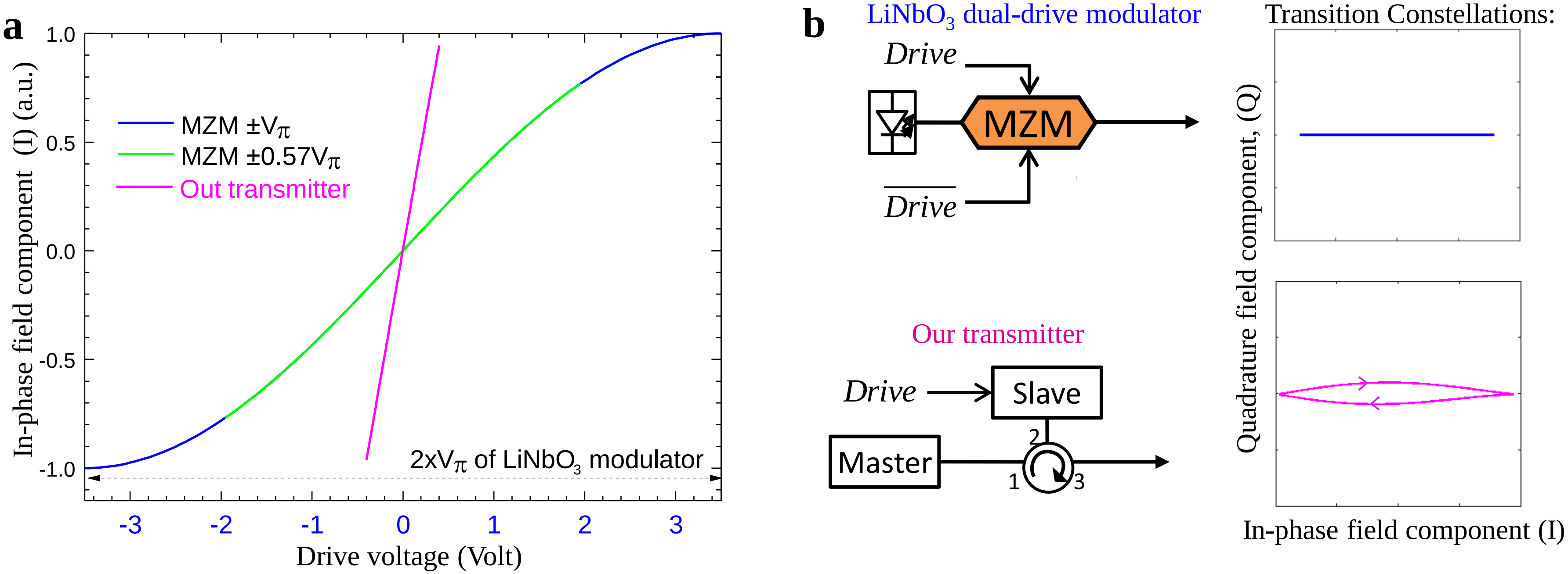

Supplementary Fig.1 a. Transmitter field response characteristics, b. Comparison of full field response of the modulators.

Supplementary Fig.1a compares the performance of a state-of-the-art commercial $LiNbO_3$ dual-drive modulator driven at $\pm V_\pi$ and $\pm 0.57V_\pi$ with our direct modulation approach (schematics of both configurations are shown in 'b'). Note in a $LiNbO_3$ IQ modulator, two such modulators are nested inside a Mach-Zehnder interferometer. To improve the linearity of the $LiNbO_3$ modulator response a narrower modulation range ($\pm 0.57V_\pi$ in our experiments) is often used in practice, resulting in an increase in insertion loss (2 dB or more). By contrast our transmitter has inherently good linearity and needs significantly lower drive voltage.

Supplementary Fig.1b shows constellation diagrams of the full field response of the modulator. For the $LiNbO_3$ modulator, there is only an in-phase field component, providing zero-chirp operation. In our Transmitter, the presence of the residual chirp manifests itself in the quadrature component, which is a common feature of all 'non-$LiNbO_3$-based' modulators currently under research.

Quantitative characterization of Linearity: Spurious-free Dynamic Range Analysis[1]

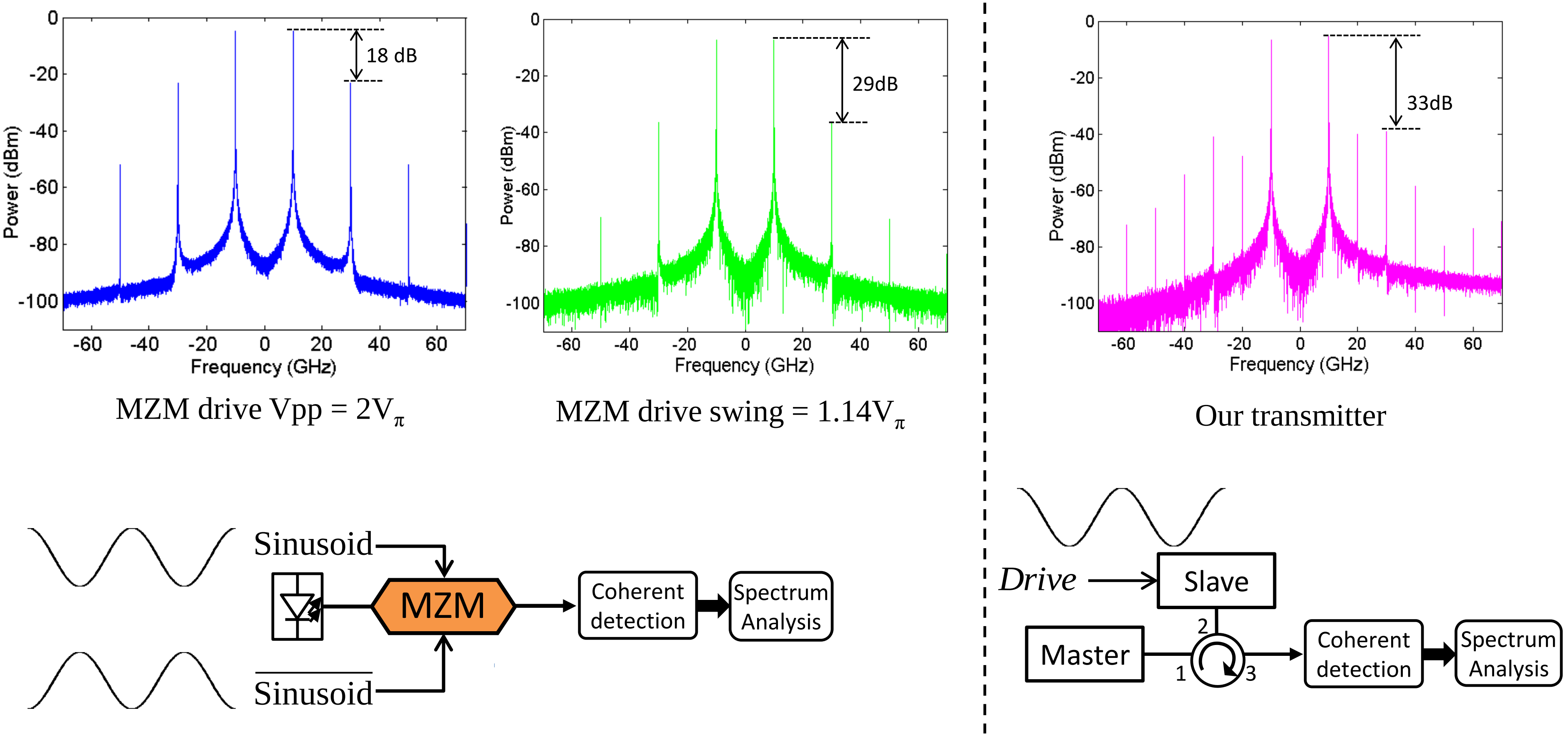

Supplementary Fig. 2. Characterization of linearity using spurious-free dynamic range (SFDR).

The spurious-free dynamic range (SFDR)[1] of our $LiNbO_3$ MZM (Mach-Zehnder Modulator) with full swing ($V_{pp} = 2V_\pi$), MZM with 57% swing ($V_{pp} = 1.14V_\pi$), and our transmitter is 18 dB, 29 dB, and 33 dB respectively, highlighting the significance of the inherent linear response of our transmitter.

1. H. Yamazaki et al., "Linear Optical IQ Modulator for High-Order Multilevel Coherent Transmission," in OFC/NFOEC, paper OM3C.1, 2013.

Generating QAM signals from binary-only RF-data streams

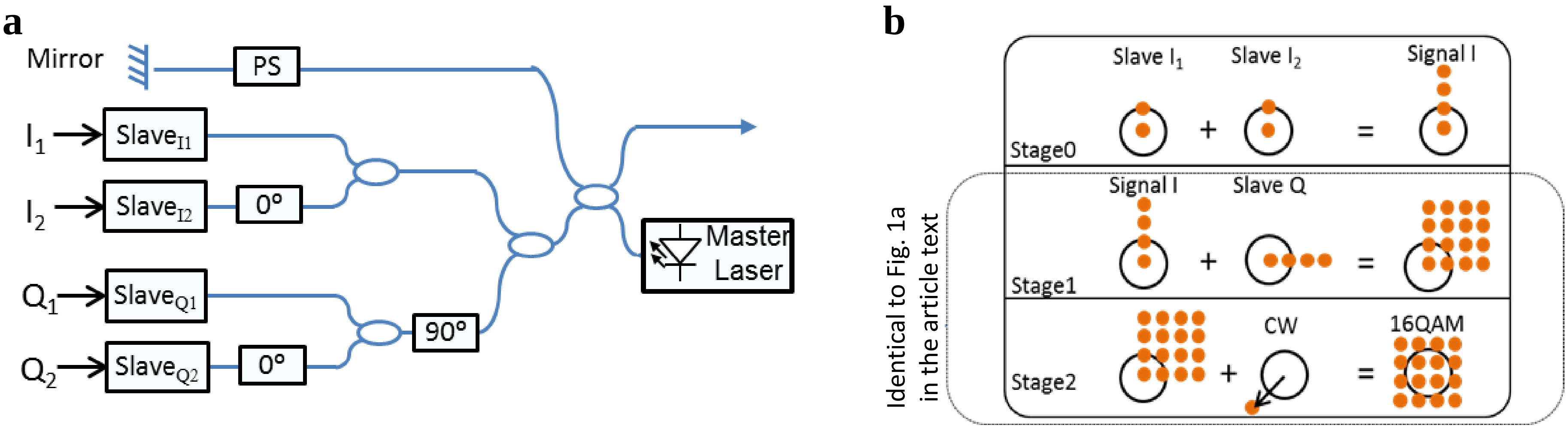

Supplementary Fig. 3. Implementation of 16QAM using four slave lasers. a. Set-up of the transmitter, b. Principle of operation shown using constellation diagrams.

Here we show how our scheme could be used to multiplex multilevel I and Q signals directly in the optical domain, requiring only binary RF data streams at its input, thereby reducing the requirement on the high speed RF electronics in terms of effective number of bits (ENOB), linearity, and loss[2].

In Supplementary Fig.3, we show schematically an example for the generation of 16QAM, although higher QAM can be obtained by using more slave lasers (for a N-bit/symbol modulation format, N lasers are required). As compared to the schematic in Fig. 1 of the article text, only a single additional step ('Stage 0') is necessary.

2. R. Slavík, et al., "Optical and RF Power Requirements for a New Injection-Locked Semiconductor Laser Diode Method Compared with Conventional Approaches for QPSK and QAM Modulations," in ECOC'13, paper P.3.22, 2013.

A ‘push-pull’ embodiment

a Implementation (16 QAM example):

b Push-pull configuration to generate 16QAM

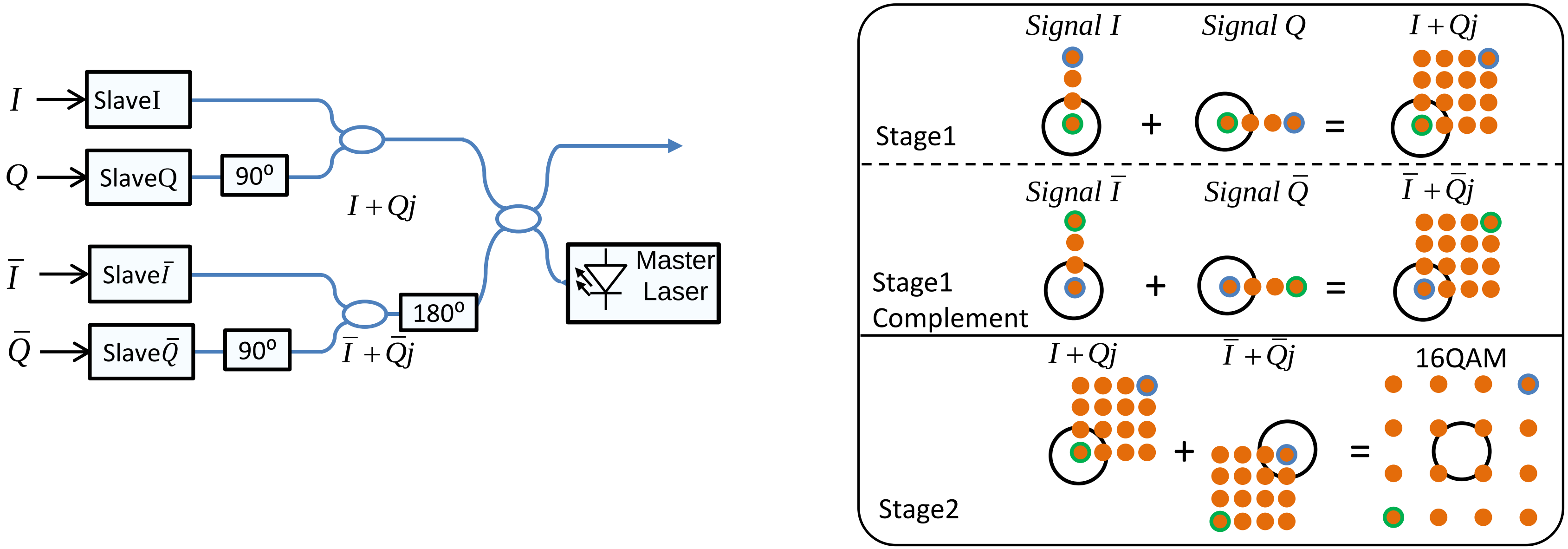

Supplementary Fig. 4 Implementation of our transmitter in push-pull operation. a. Set-up of the our transmitter, b. Principle of operation shown using constellation diagrams.

Our transmitter concept can also support push-pull operation, which cancels the carrier directly and may result in lower insertion loss and even lower RF power requirement. The output signal-to-noise ratio is increased by a factor of two in the process.

Supplementary Fig.4 shows the principle applied to the case of 16QAM. First, we generate the signal as inthe previous slide (Stages 0+1), shown as Stage 1 in ‘b’. Then, we generate a complementary signal (shown as Stage 1 Complement that has the same constellation, but for which the data is encoded complementarily (e.g., a green dot in ‘Stage 1’ is on the bottom-left edge of the constellation, while the same data point is now at the top-right edge on the ‘Stage 1 Complement’ constellation). By destructively combining the two complementary data streams, we obtain a carrier-less 16QAM signal, as shown schematically as Stage 2. The schematic is shown in ‘a’.
